# Decreased bone mineral density is associated with an increased number of teeth with periodontitis progression: a 5-year retrospective cohort study

**DOI:** 10.1007/s00784-023-05463-8

**Published:** 2023-12-28

**Authors:** Phanthapha Khunthananithi, Attawood Lertpimonchai, Chanika Sritara, Supreda Suphanantachat Srithanyarat, Lalitsara Thienpramuk, Sanutm Mongkornkarn

**Affiliations:** 1 Department of Periodontology, Faculty of Dentistry, Chulalongkorn University, 34 Henri Dunant Road, Wangmai, Pathumwan, Bangkok, 10330 Thailand; 2 Center of Excellence in Periodontal Disease and Dental Implant, Chulalongkorn University, Bangkok, Thailand; 3 Division of Nuclear Medicine, Department of Diagnostic and Therapeutic Radiology Faculty of Medicine Ramathibodi Hospital, Mahidol University, Bangkok, Thailand; 4 Medical and Health Department, Health Division, Electricity Generating Authority of Thailand, Nonthaburi, Thailand

**Keywords:** Bone density, Longitudinal, Osteoporosis, Periodontal disease, Periodontitis

## Abstract

**Objective:**

Longitudinal studies on the systemic bone loss-periodontitis relationship are limited with disparate results. The aim of this study was to investigate the association between bone mineral density (BMD) and periodontitis progression, controlling for other covariables in a Thai population.

**Materials and methods:**

In 2,418 participants, BMD values of the lumbar spine, femoral neck, and total hip were measured with dual-energy X-ray absorptiometry at baseline. Each participant’s BMD status was classified as normal, osteopenia, or osteoporosis. Full mouth periodontal examinations on 6 sites/tooth were performed at baseline and 5-year follow-up visits. Periodontitis progression was defined as a tooth presenting an additional proximal CAL loss of ≥ 3 mm or an additional lost tooth with a baseline CAL ≥ 5 mm. The risk effects of BMD status on the number of teeth with periodontitis progression were analyzed using multivariate Poisson regression.

**Results:**

Baseline BMD status of osteoporosis was associated with an increased number of teeth with periodontitis progression in the subgroups of postmenopausal women, non-smokers, and participants with periodontitis stage III/IV with adjusted risk ratios of 1.31 (95% CI = 1.09–1.58), 1.19 (95% CI = 1.04–1.36), and 1.13 (95% CI = 1.00–1.28), respectively.

**Conclusion:**

Baseline BMD in the osteoporosis range increased the risk of having a greater number of teeth with periodontitis progression in specific participant subgroups.

**Clinical Relevance:**

Decreased BMD is a potential factor affecting periodontitis progression risk in some individuals. Multidisciplinary approaches in educating and maintaining patients’ bone-oral health may help improve their quality of life.

**Supplementary Information:**

The online version contains supplementary material available at 10.1007/s00784-023-05463-8.

## Introduction

Periodontitis is a chronic inflammation of the periodontium that destroys the bone, periodontal ligament, and cementum. It is regarded as the main cause of tooth loss and affects patients’ quality of life [1, 2]. Periodontitis progression is associated with a host-microbiome dysbiosis [1] and modified by several local, systemic, and genetic factors [3, 4]. Osteoporosis is a manifestation of systemic bone loss due to decreased bone mineral density (BMD), leading to bone fragility [5]. This bone disorder is potentially related to periodontitis because they are both associated with several common factors, e.g., age, sex, diabetes, smoking, alcohol consumption, body size, and socioeconomic status [6, 7]. Moreover, increased inflammatory cytokine levels related to bone resorption [8] may be the biological link between osteoporosis and periodontal destruction[6, 7, 9, 10], as supported by a correlation between systemic bone loss and oral bone loss [11–13]or periodontal inflammation [14].

Currently, the relationship of systemic bone loss and periodontitis is unresolved [15], with limited numbers of longitudinal studies to confirm the causal relationship between these two diseases [11, 12, 14, 16–19]. Although several studies found an association between systemic bone loss or decreased BMD and periodontitis as measured by alveolar bone loss [11, 12], clinical attachment (CAL) loss [17, 19], or tooth loss [16, 18, 19], others failed to show a relationship between the two diseases [20–22]. These inconsistent findings may be due to different study populations, periodontitis case definitions used, and methods of variable or bone density measurements. Moreover, most previous studies were conducted in postmenopausal women. Therefore, the aim of this retrospective cohort study was to investigate the association between BMD status and periodontitis progression, controlling for known confounders in Thai adults and elders comprising a larger sample size with a wide age range, covering both sexes, using standard methods of variable measurements and case definitions. The data from this study will broaden the knowledge of a possible causal relationship between these two diseases.

## Material and methods

### Study sample and data collection

This cohort study was conducted in current and ex-employees of the Electricity Generating Authority of Thailand (EGAT) in accordance with the Strengthening the Reporting of Observational Studies in Epidemiology (STROBE) guidelines. The EGAT cohort study profiles have been previously described [23]. The study protocol was approved by the Ethics Committee of the Faculty of Dentistry, Chulalongkorn University (HREC-DCU 2021–114) and the Faculty of Medicine Ramathibodi Hospital, Mahidol University (COA.MURA2022/377), Thailand.

All participants signed informed consents before each survey and received advice about their treatment needs after the surveys. Baseline data, including BMD values and dental data, were obtained from the previous cross-sectional study [24] that was performed on the participants (30–82 years old) involved in the 2012 (EGAT 1/5) and the 2014 (EGAT 3/2) surveys. The participants who required antibiotic premedication before a dental examination [25], or with conditions that potentially affected bone metabolism or DXA analysis as previously specified [26] were excluded. Five years later, the participants from the previous two surveys were consecutively enrolled in the 2017 (EGAT 1/6) and the 2019 (EGAT 3/3) surveys, and their dental data from these follow-up visits were analyzed for the association of baseline BMD status and periodontitis progression.

### BMD assessment

At baseline, the participants underwent BMD assessment of the lumbar spine (L1–L4 vertebrae), femoral neck, and total hip using dual-energy X-ray absorptiometry (DXA).^1^ The details of the measurements were previously described [27]. In the present study, the main independent variable was the baseline BMD status calculated from the worst-site BMD-score [24], using reference values from non-Hispanic white women aged 20–29 years old [28]. The participants were classified into three BMD groups of normal, osteopenia, or osteoporosis when their BMD T-scores were within -1 SD, between -1 SD and -2.5 SD, and ≤ -2.5 SD of the reference values, respectively [24, 29].

### Periodontal assessment

The participants underwent periodontal assessment at baseline and follow-up visits by the same group of 8 calibrated periodontists from the Department of Periodontology, Faculty of Dentistry, Chulalongkorn University in mobile dental units. The dental examination and calibration details were reported in previous EGAT studies [24, 25]. Weighted kappa (± 1 mm) was used to determine the inter-examiner and intra-examiner agreements (Supplementary Table 1). The periodontal assessments comprised of records of missing teeth, presence of supragingival plaque by wiping a probe across 2 sites/tooth, and measuring probing depth (PD) and gingival recession (RE) on 6 sites/tooth with a periodontal probe.^2^ The modified plaque score was determined as previously described [30], and CAL was calculated from the RE and PD [24, 25]. The participants’ baseline periodontal status were categorized based on the 2018 AAP/EFP periodontitis classification [31] and used as one of the covariables in the Poisson regression analyses.

### Outcome variable

The primary outcome of this study was the number of teeth with periodontitis progression at the patient level. The criteria for periodontitis progression at the tooth level were described in the previous EGAT study [32] defined as a tooth presenting an additional proximal CAL loss of ≥ 3 mm or an additional lost tooth with baseline proximal CAL ≥ 5 mm (severe periodontitis).

## Statistical analysis

The statistical analyses were performed using a standard software,^3^ and significance was considered at *P* < 0.05. For data analysis, the baseline characteristics were described by mean ± one SD for continuous data, and by frequency and percentage for categorical data. The difference in changes in mean CAL, mean PD, and mean number of teeth with periodontitis progression at the 5-year follow-up were compared between the BMD statuses by one-way analysis of variance (ANOVA) and Bonferroni post-hoc test. The associations between BMD status and the number of teeth with periodontitis progression were analyzed using Poisson regression analysis. The analyses were performed in the whole study population, the subgroups of postmenopausal woman, participants with different smoking status and baseline periodontitis severities. The covariables considered in the regression model were age, sex, baseline periodontitis stage, plaque score, self-reported periodontal treatment, diabetes, body mass index (BMI), smoking, alcohol consumption, income, education, and the use of medication related to bone. The covariables with a *P*-value < 0.1 in the univariate analysis and known variables risk for periodontitis progression were considered in the multivariate analysis using the forward method of variable selection. Risk ratios (RRs) and their 95% confidence intervals (CIs) were estimated.

## Results

**Fig. 1:**
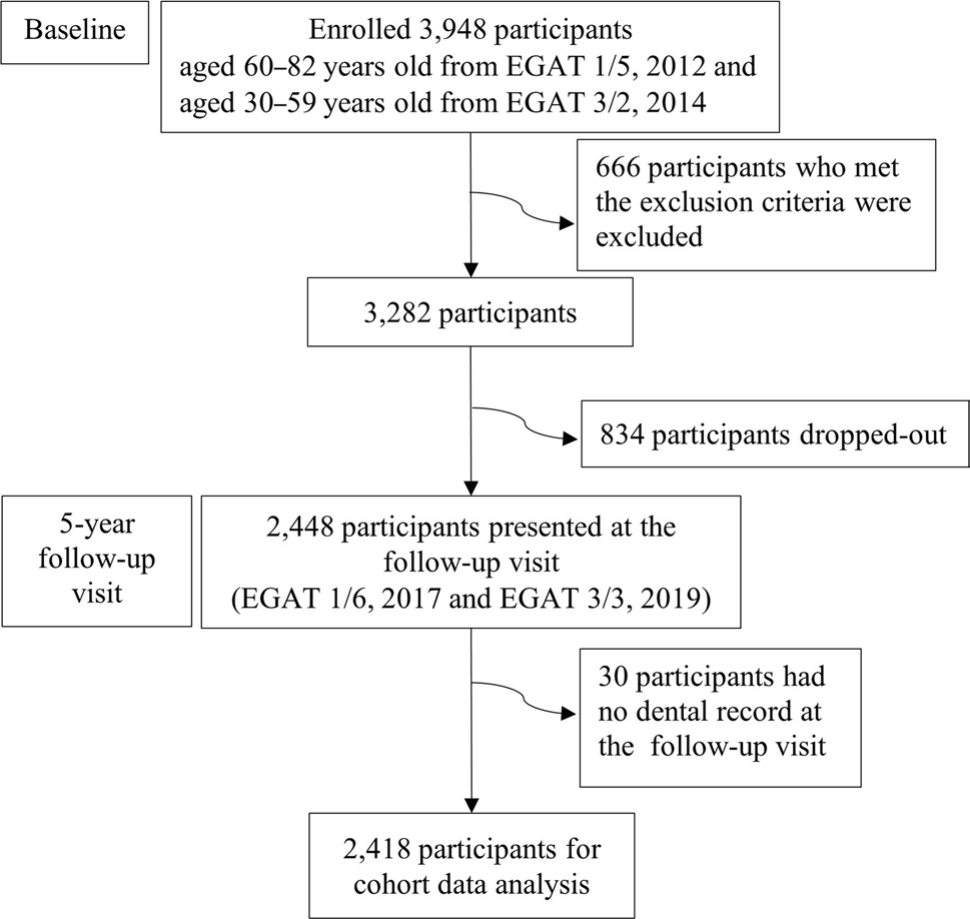
Flow of the study participants

The flow of our study participants is shown in Fig. 1. Of the 3,282 participants that were included from the baseline surveys, 2,448 participants presented for the 5-year follow up. After excluding 30 participants who had no dental record, the data of 2,418 participants who were at the baseline and the follow-up visits were analyzed for the association between baseline BMD status and periodontitis progression.

The participants’ baseline characteristics are shown in Table 1. In the whole study population, their mean age was 52.6 ± 12.0 years old and 69.9% were males. According to the 2018 AAP-EFP classification, the periodontitis prevalence was 66.7%, and 49.5% of the participants had periodontitis stage III/IV. Most participants had poor oral hygiene as reflected by their mean plaque score of 64.8%, despite most of them (87.1%) reporting having periodontal treatment. Approximately two-thirds of the participants were overweight or obese, however, most were non-diabetes (91.4%). Only 4.4% of the participants were heavy smokers and 12.6% were frequent drinkers. Regarding BMD status, 42% of the participants had normal bone, while 50% and 8% were classified as osteopenia and osteoporosis, respectively.

**Table 1:** Baseline characteristics of the study participants

Baseline characteristics	Whole study population (n = 2,418)	Postmenopausal women (n = 305)
Age (years), mean ± SD	52.6 ± 12.0	60.3 ± 8.7
Sex		
Female	728 (30.1)	305 (100)
Male	1,690 (69.9)	0 (0)
Baseline periodontal status (AAP/EFP 2018)		
No	806 (33.3)	111 (36.4)
Stage I	1 (0)	0 (0)
Stage II	416 (17.2)	40 (13.1)
Stage III/IV	1196 (49.5)	154 (50.5)
Plaque score (%), mean ± SD	64.8 ± 21.8	62.2 ± 21.6
< 40%	297 (12.3)	69 (22.6)
40–79%	1,460 (60.4)	197 (64.6)
≥ 80%	661 (27.3)	39 (12.8)
CAL (mm), mean ± SD	2.68 ± 0.92	2.64 ± 0.82
PD (mm), mean ± SD	2.30 ± 0.50	2.21 ± 0.49
Tooth loss, mean ± SD	6.4 ± 5.3	7.7 ± 5.5
Periodontal treatment (self-reported)^a^		
No	311 (12.9)	30 (9.9)
Yes	2,098 (87.1)	274 (90.1)
BMD status		
Normal	1,017 (42.0)	99 (32.5)
Osteopenia	1,196 (49.5)	166 (54.4)
Osteoporosis	205 (8.5)	40 (13.1)
HbA1c (%), mean ± SD	5.7 ± 0.8	5.9 ± 0.8
Diabetes mellitus^b^		
No	2,210 (91.4)	272 (89.2)
Yes	208 (8.6)	33 (10.8)
BMI (kg/m^2^), mean ± SD	24.7 ± 3.7	24.1 ± 4.2
Underweight (< 18.5)	67 (2.8)	14 (4.6)
Normal (18.5–22.9)	756 (31.3)	117 (38.4)
Overweight (23–24.9)	575 (23.7)	65 (21.3)
Obese (≥ 25)	1,020 (42.2)	109 (35.7)
Smoking status^c^		
Non-smoker	1,564 (64.9)	297 (97.4)
Former smoker	619 (25.7)	7 (2.3)
Light smoker (< 10 cigarettes/day)	120 (5.0)	1 (0.3)
Heavy smoker (≥ 10 cigarettes/day)	106 (4.4)	0
Alcohol consumption^d^		
Non-drinker	1,816 (75.2)	299 (98.0)
Occasional drinker (< 1 time/week)	295 (12.2)	5 (1.7)
Frequent drinker (≥ 1 time/week)	305 (12.6)	1 (0.3)
Income (USD/month)		
< 600	469 (19.4)	71 (23.3)
600–1,499	713 (29.5)	96 (31.5)
≥ 1,500	1,236 (51.1)	138 (45.2)
Education		
< High school	148 (6.1)	21 (6.9)
Diploma	481 (19.9)	59 (19.3)
Bachelor or higher	1,789 (74.0)	225 (73.8)
Menopause, mean age ± SD	47.9 ± 5.6	47.9 ± 5.6
Medication related to bone^e^		
No	2,264 (93.6)	235 (77.0)
Yes	154 (6.4)	70 (23.0)

Abbreviations: *BMD*, bone mineral density; *BMI*, body mass index; *CAL*, clinical attachment level; *HbA1c*, glycated hemoglobin; *PD*, probing depth; *SD*, standard deviation

^a^Missing the data of 9 participants, the treatment comprised of scaling, root planing, and periodontal surgery

^b^Diabetes was diagnosed based on the participants' medical history of fasting plasma glucose levels of ≥ 126 mg/dl or the use of any type of anti-diabetic medication

^c^Missing the data of 9 participants

^d^Missing the data of 2 participants

^e^Medication related to bone were vitamin D, calcium, and hormone replacement

Postmenopausal women accounted for 12.6% of the whole study population, or 42% of the females. This group had a mean age of 60.3 ± 8.7 years old, with baseline characteristics consistent with the whole study population, except for a lower prevalence of current smokers (0.3%), greater prevalence of osteopenia (54%) or osteoporosis (13%), and greater proportion of participants using medication related to bone (23%) (Table 1).

Changes in the mean periodontal variables of the whole study population and postmenopausal women according to their bone status at the 5-year follow-up were compared using ANOVA and Bonferroni post-hoc tests (Table 2). The mean number of teeth with periodontitis progression of the whole study population and the postmenopausal subgroup was 2.0 ± 3.0 and 1.7 ± 2.4, respectively. In postmenopausal women, the osteoporosis group had the greatest mean number of teeth with periodontitis progression. We found a significant increase in the mean number of teeth (mean increase of 0.9 teeth) with periodontitis progression in the osteoporosis group compared with the osteopenia group. In addition to the pooled results, we also found the mean number of tooth loss significantly increased as the bone status worsened.

**Table 2:**
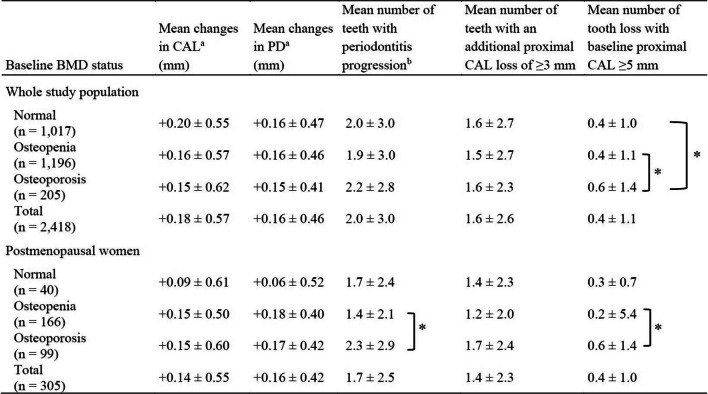
Changes in the mean periodontal variables according to bone status at the 5-year follow-up of the whole study population and postmenopausal women (mean ± SD)

Abbreviations: *BMD*, bone mineral density; *CAL*, clinical attachment level; *PD*, probing depth; *SD*, standard deviation

^a^Positive values represented an increase in CAL loss/PD

^b^Periodontitis progression was defined as a tooth presenting an additional proximal CAL loss of ≥ 3 mm or an additional lost tooth with a baseline proximal CAL ≥ 5 mm

Differences in periodontal parameters between bone status groups were analyzed using one-way ANOVA and Bonferroni post-hoc test

^*^Significant difference (*P* < 0.05)

**Table 3:** Risk ratios and 95% confidence intervals for an increased number of teeth with periodontitis progression in the whole study population and various population subgroups

Baseline characteristics	Adjusted RR (95% CI) for an Increased Number of Teeth with Periodontitis Progression^a^						
	Whole study population (n = 2,400)	Population subgroups					
		Postmenopausal women (n = 304)	Non-smokers (n = 1,561)	Current/Former Smokers (n = 839)	Periodontitis stage		
					No/stage I (n = 803)	Stage II (n = 415)	Stage III/IV (n = 1,182)
BMD status							
Normal/osteopenia	1	1	1	1	1	1	1
Osteoporosis	1.08 (0.97–1.19)	1.31 (1.09–1.58)^*^	1.19 (1.04–1.36)^*^	0.92 (0.77–1.10)	1.05 (0.84–1.31)	0.70 (0.47–1.06)	1.13 (1.00–1.28)^*^
Sex							
Female	1	N/A	1	1	1	1	1
Male	1.27 (1.17–1.38)^*^		1.32 (1.21–1.44)^*^	1.14 (0.86–1.51)	1.27 (1.08–1.49)^*^	1.23 (1.00–1.50)^*^	1.25 (1.12–1.40)^*^
Age (1 year increment)	1.01 (1.01–1.02)^*^	1.03 (1.02–1.05)^*^	1.01 (1.01–1.02)^*^	1.01 (1.00–1.01)^*^	1.03 (1.02–1.04)^*^	1.02 (1.01–1.03)^*^	1.01 (1.00–1.01)^*^
Periodontal status (AAP/EFP 2018)							
No/stage I	1	1	1	1	N/A	N/A	N/A
Stage II	1.22 (1.10–1.36)^*^	1.28 (0.93–1.76)	1.33 (1.17–1.51)^*^	0.99 (0.82–1.20)			
Stage III/IV	1.77 (1.64–1.92)^*^	1.74 (1.41–2.16)^*^	1.73 (1.57–1.91)^*^	1.83 (1.61–2.07)^*^			
Plaque score							
< 40%	1	1	1	1	1	1	1
40%**–**79%	1.32 (1.18–1.47)^*^	1.24 (0.93–1.82)	1.39 (1.20–1.61)^*^	1.22 (1.02–1.47)^*^	1.20 (0.99–1.45)	1.54 (1.15–2.01)^*^	1.36 (1.16–1.60)^*^
≥ 80%	1.45 (1.29–1.63)^*^	1.32 (0.95–1.82)	1.59 (1.36–1.85)^*^	1.36 (1.13–1.64)^*^	1.49 (1.19–1.85)^*^	1.09 (0.78–1.53)	1.54 (1.31–1.81)^*^
Diabetes mellitus^b^							
No	1	1	1	1	1	1	1
Yes	1.08 (0.98–1.18)	0.97 (0.73–1.30)	1.00 (0.87–1.15)	1.16 (1.02–1.31)^*^	1.07 (0.84–1.35)	1.38 (1.04–1.82)^*^	1.05 (0.94–1.17)
Smoking status							
Non-smoker	1	1	N/A	N/A	1	1	1
Former smoker	1.12 (1.04–1.21)^*^	1.12 (0.68–1.83)			1.15 (0.97–1.37)	0.69 (0.53–0.89)^*^	1.19 (1.09–1.30)^*^
Light smoker	1.38 (1.22–1.56)^*^	0.67 (0.21–2.15)			1.32 (0.93–1.87)	0.92 (0.66–1.27)	1.48 (1.28–1.70)^*^
Heavy smoker	1.60 (1.43–1.79)^*^	N/A			1.09 (0.58–2.05)	2.05 (1.51–2.78)^*^	1.56 (1.38–1.77)^*^
Self-report periodontal treatment							
Yes	1	1	1	1	1	1	1
No	1.19 (1.10–1.28)^*^	0.86 (0.65–1.14)	0.95 (0.84–1.08)	1.42 (1.29–1.56)^*^	0.99 (0.82–1.20)	1.54 (1.11–2.13)^*^	1.20 (1.10–1.31)^*^
Education							
≥ Bachelor’s degree	1	1	1	1	1	1	1
< Bachelor’s degree	1.29 (1.21–1.37)^*^	1.19 (0.98–1.44)	1.50 (1.36–1.64)^*^	1.10 (1.01–1.20)^*^	1.08 (0.92–1.28)	1.17 (0.94–1.45)	1.33 (1.23–1.43)^*^

Abbreviation: *BMD*, bone mineral density

^a^Risk ratios and 95% confidence intervals were obtained using Poisson regression analysis

^b^Diabetes was diagnosed based on the participants' medical history of fasting plasma glucose levels of ≥ 126 mg/dl or the use of any type of anti-diabetic medication

^*^Significant difference (*P* < 0.05)

The risk effects of bone status on an increased number of teeth with periodontitis progression were analyzed using Poisson regression (Table 3). In the whole study population, the univariate analysis revealed that osteoporosis was associated with a greater number of teeth with periodontitis progression with unadjusted RRs of 1.13 (95% CI = 1.03–1.25) (data not shown). However, after adjusting for confounders, the result was no longer significant. When analyzing the data in various population subgroups, significant associations between osteoporosis and an increased number of teeth with periodontitis progression were demonstrated in postmenopausal women, non-smokers, and participants with baseline periodontitis stage III/IV with adjusted risk ratios of 1.31 (95% CI = 1.09–1.58), 1.19 (95% CI = 1.04–1.36), and 1.13 (95% CI = 1.00–1.28), respectively. In addition to the pooled results, we also found that osteoporosis was associated with an increased number of tooth loss in the whole study population, subgroups of postmenopausal women, and non-smokers (Supplementary Table 2).

## Discussion

This study demonstrated that baseline BMD in the osteoporosis range was associated with an increased number of teeth with periodontitis progression at the 5-year follow-up among postmenopausal women, non-smokers, and individuals with baseline periodontitis stage III/IV. Although these significant findings of BMD status-associated periodontal disease concurred with several previous longitudinal studies [11, 12, 14, 16–19], they contrasted with others [20–22], These differences may be due to differences in sample size and follow-up times compared with our study.

## Prevalence of osteoporosis and periodontitis progression

The prevalence of osteoporosis is related to increasing age and is more common in women [33]. Therefore, the prevalence of osteoporosis in our study that included young participants that were mostly males was lower than that reported in the US adults aged ≥ 50 years [33] (8.5% vs. 12.6%). In the postmenopausal women subgroup, the osteoporosis prevalence increased to 13%, and was consistent with a survey that used the NHANES data references [33].

The incidence of periodontitis progression depends on the case definitions, follow-up period, access to dental care, and maintenance protocol. The definition of teeth that demonstrated periodontitis progression used in this study was modified from previous reports [32, 34]. To reduce the underestimation of periodontitis progression, additional proximal CAL loss of ≥ 3 mm and tooth loss with baseline CAL ≥ 5 mm were included in the criteria. Although CAL loss of ≥ 2 mm in 5 years is considered periodontitis grade C (rapid progression) [31], a ≥ 3 mm CAL loss threshold was recommended in risk factor research to compensate for an error of 2.5 mm from the recording method [34], and because most of our study participants (~ 90%) fell into periodontitis grade C. Using these criteria in this EGAT population, in which most of them received periodontal care (self-reported), 48.7% of the participants had ≥ 1 teeth with periodontitis progression at the 5-year follow-up. Limited access to dental care increased periodontitis progression as shown in a 4-year study of a rural Chinese population [35] that reported a 68% incidence rate when using only ≥ 1 teeth with ≥ 3 mm proximal CAL loss to define disease progression.

## Changes in mean periodontal variables

In our study, the mean CAL and PD changes at the 5-year follow-up were not significantly different between bone status groups, thus, the changes in the whole-mouth mean values may not reflect disease progression at specific sites. These findings concurred with previous studies that found no significant increase in mean CAL loss in individuals who had worse bone status [12, 14]. However, in the postmenopausal women subgroup, our finding that the osteoporosis group had the greatest mean number of teeth with periodontitis progression suggested that impaired bone status may be related to the progression of periodontitis and needs further investigation. The significant difference in the mean number of teeth with periodontitis progression of ~ 0.9 teeth at the 5-year follow-up, i.e., 0.18 teeth/year, found between the osteoporosis and osteopenia groups, may be clinically relevant because these values concurred with the average mean tooth loss of ~ 0.2 teeth/year reported in a meta-analysis of prospective studies on the progression of periodontitis in the general population [36].

## Risk effects of BMD status on periodontitis progression

In the whole study population, the multivariate Poison regression analysis revealed that baseline BMD status was not associated with an increased risk of having a greater number of teeth with periodontitis progression. This finding suggests that other variables have a greater impact than that of BMD status on periodontitis progression. However, in the subgroup analyses of postmenopausal women and non-smokers, the findings that those with baseline BMD status of osteoporosis had an ~ 1.2–1.3 fold higher likelihood of having a greater number of teeth with periodontitis progression than non-osteoporosis individuals concurred with the previous studies that reported associations between worse BMD and periodontitis progression in postmenopausal women [11, 12, 16, 18, 19], non-smokers [11, 17–19] or populations with a low prevalence of current smokers [12, 16]. Because 97% of the postmenopausal women were non-smokers, and the influence of male sex [25] and smoking [37, 38] were excluded in the subgroup analyses, the effect of osteoporosis on periodontitis progression then became apparent in these population subgroups. The protective effect of medications related to bone on periodontitis progression and tooth loss was not found in our study but has been previously reported [39–41]. As medications related to bone is mainly calcium supplements, the limited number of participants (< 3%) who used hormone or vitamin D supplements might explain the incongruity to the previous studies.

In the subgroup of participants with baseline periodontitis stage III/IV, our finding that osteoporosis was associated with a 13% increased risk of having a greater number of teeth with periodontitis progression is interesting. This finding suggests that considering decreased BMD as another potential factor associated with an increased risk of periodontitis progression in severe periodontitis individuals, in addition to the commonly known factors that were reported in the literature and were also confirmed in our study e.g., smoking [37, 38], being male [25], increased age [38, 42], poor oral hygiene [25], no periodontal treatment [43], and low educational level [44]. That diabetes was not associated with periodontitis progression in most of our participant subgroups concurred with the previous EGAT studies [24, 32], and is likely because most of our participants had relatively good glycemic control [45], and is supported by a study where well-controlled diabetes did not increase the risk of periodontitis progression or tooth loss [46].

A meta-analysis on the relationship between systemic bone loss and periodontitis revealed that osteoporosis patients had an increased risk of periodontitis (OR = 1.96; 95% CI = 1.50–2.54)[47]. However, the causative effect of bone status on periodontitis progression was disparate among cohort studies. However, our results concurred with most studies that reported a significant association between BMD and periodontitis progression, mainly in postmenopausal women [11, 12, 14, 16, 18, 19]. Currently, there is only one prospective study [17] conducted in both sexes where they found that low BMD was associated with an increase in the number of progressive sites (≥ 3 mm additional proximal CAL loss) in 3 years. However, this study had a small sample size with the participants’ age limited to 70 years old and used ultrasound densitometry as a community screening for heel BMD. Other than CAL, decreased BMD was reported to be associated with reduced alveolar bone height [11, 12] or an increased number of tooth loss [16, 18]. With risk effects similar to those in our results, a study of non-smoking postmenopausal women [18] reported a 1.2–1.4-fold increase in the risk of tooth loss in women with the third tertile vs. the first tertile of annual decrease in BMD.

In contrast with our findings and the studies mentioned above, other cohort studies did not demonstrate a significant association between bone status and periodontitis progression[20–22]. The non-significant findings may partly be explained by their limited sample sizes and age-ranges, and only posterior teeth or half mouths were examined, which may underestimate the incidence of periodontitis progression; and follow-up times of less than 3-years that may be insufficient to observe periodontitis progression.

## Strengths and limitations

Our study strengths were the large sample size of wide age ranges covering both sexes, a long follow-up time, full-mouth periodontal assessments by calibrated periodontists, BMD assessment at 3 skeletal sites with the current standard method, i.e., DXA analysis [29], and adequate control of confounders in data analyses. However, this study has some limitations. Dental radiographs, which can be additional evidence of periodontitis progression, were not available. The reasons for tooth loss were not recorded, therefore, baseline periodontitis stage III and IV were combined, and we only counted a tooth loss with baseline CAL ≥ 5 mm to minimize overestimating tooth loss due to periodontitis progression. Due to the limitation of self-reported questionnaire regarding definite active and supportive periodontal treatments, past periodontal treatment can only be categorized as “yes/no” for confounder adjustment. No BMD data at the follow-up visit restricted the analysis of the influence of changes in BMD status on periodontal disease. Moreover, this study was conducted in the EGAT population, therefore, the results still need to be confirmed in additional populations. Finally, because skeletal BMD may be affected by calcified degenerative changes [48, 49] trabecular bone score, another bone index for grading bone quality, should also be analyzed for systemic bone loss associated periodontitis progression.

## Conclusion

This study demonstrated that BMD that decreased to the osteoporosis range increased the risk of having a greater number of teeth with periodontitis progression in postmenopausal women, non-smokers, and those with periodontitis grade III/IV in the EGAT population. These findings suggest the benefit of monitoring bone status as another potential factor affecting periodontitis progression. Multidisciplinary approaches in educating and maintaining ones’ bone-oral health may help improve their quality of life.

### Supplementary Information

Below is the link to the electronic supplementary material.

Supplementary file1 (DOCX 31 KB)

Supplementary file2 (DOCX 35 KB)

## Footnotes

1 Discovery QDR 4500W, Hologic, Bedford, MA, USA.

2 PCP-UNC15 periodontal probe.

3 Statistic Package for the Social Science (SPSS, Chicago, USA) version 28.0.

## References

[1] Hajishengallis G (2014). Immunomicrobial pathogenesis of periodontitis: keystones, pathobionts, and host response. Trends Immunol.

[2] Ferreira M, Dias-Pereira A, Branco-de-Almeida L, Martins C, Paiva S (2017). Impact of periodontal disease on quality of life: a systematic review. J Periodontal Res.

[3] Kornman KS (2008). Mapping the pathogenesis of periodontitis: a new look. J Periodontol.

[4] Kinane DF, Stathopoulou PG, Papapanou PN (2017). Periodontal diseases. Nat Rev Dis Primers.

[5] Klibanski A, Adams-Campbell L, Bassford T (2001). Osteoporosis prevention, diagnosis, and therapy. J Am Med Assoc.

[6] Guiglia R, Di Fede O, Lo Russo L, Sprini D, Rini GB, Campisi G (2013). Osteoporosis, jawbones and periodontal disease. Med Oral Patol Oral Cir Bucal.

[7] Wang CJ, McCauley LK (2016). Osteoporosis and periodontitis. Curr Osteoporos Rep.

[8] Barbour KE, Lui LY, Ensrud KE (2014). Inflammatory markers and risk of hip fracture in older white women: the study of osteoporotic fractures. J Bone Miner Res.

[9] Lerner U (2006). Inflammation-induced bone remodeling in periodontal disease and the influence of post-menopausal osteoporosis. J Dent Res.

[10] Yu B (2000). Wang CY (2022) Osteoporosis and periodontal diseases-an update on their association and mechanistic links. Periodontol.

[11] Payne JB, Reinhardt RA, Nummikoski PV, Patil KD (1999). Longitudinal alveolar bone loss in postmenopausal osteoporotic/osteopenic women. Osteoporos Int.

[12] LaMonte MJ, Hovey KM, Genco RJ, Millen AE, Trevisan M, Wactawski-Wende J (2013). Five-year changes in periodontal disease measures among postmenopausal females: the Buffalo OsteoPerio study. J Periodontol.

[13] Wactawski-Wende J, Hausmann E, Hovey K, Trevisan M, Grossi S, Genco RJ (2005). The association between osteoporosis and alveolar crestal height in postmenopausal women. J Periodontol.

[14] Pereira FM, Rodrigues VP, de Oliveira AE, Brito LM, Lopes FF (2015). Association between periodontal changes and osteoporosis in postmenopausal women. Climacteric.

[15] Albandar JM, Susin C, Hughes FJ (2018). Manifestations of systemic diseases and conditions that affect the periodontal attachment apparatus: case definitions and diagnostic considerations. J Periodontol.

[16] Krall E, Garcia R, Dawson-Hughes B (1996). Increased risk of tooth loss is related to bone loss at the whole body, hip, and spine. Calcif Tissue Int.

[17] Yoshihara A, Seida Y, Hanada N, Miyazaki H (2004). A longitudinal study of the relationship between periodontal disease and bone mineral density in community-dwelling older adults. J Clin Periodontol.

[18] Iwasaki M, Nakamura K, Yoshihara A, Miyazaki H (2012). Change in bone mineral density and tooth loss in Japanese community-dwelling postmenopausal women: a 5-year cohort study. J Bone Miner Metab.

[19] Penoni DC, Leao ATT, Torres SR (2018). Effects of bone fragility and antiresorptive drugs on periodontal disease and tooth loss: a longitudinal study. JDR Clin Trans Res.

[20] Reinhardt RA, Payne JB, Maze CA, Patil KD, Gallagher SJ, Mattson JS (1999). Influence of estrogen and osteopenia/osteoporosis on clinical periodontitis in postmenopausal women. J Periodontol.

[21] Famili P, Cauley J, Suzuki J, Weyant R (2005). Longitudinal study of periodontal disease and edentulism with rates of bone loss in older women. J Periodontol.

[22] Phipps KR, Chan BK, Madden TE (2007). Longitudinal study of bone density and periodontal disease in men. J Dent Res.

[23] Vathesatogkit P, Woodward M, Tanomsup S (2012). Cohort profile: the electricity generating authority of Thailand study. Int J Epidemiol.

[24] Mongkornkarn S, Suthasinekul R, Sritara C, Lertpimonchai A, Tamsailom S, Udomsak A (2019). Significant association between skeletal bone mineral density and moderate to severe periodontitis in fair oral hygiene individuals. J Investig Clin Dent.

[25] Torrungruang K, Tamsailom S, Rojanasomsith K (2005). Risk indicators of periodontal disease in older Thai adults. J Periodontol.

[26] Sritara C, Thakkinstian A, Ongphiphadhanakul B (2016). Age-adjusted dual X-ray absorptiometry-derived trabecular bone score curve for the lumbar spine in Thai females and males. J Clin Densitom.

[27] Sritara C, Ongphiphadhanakul B, Chailurkit L, Yamwong S, Ratanachaiwong W, Sritara P (2013). Serum uric acid levels in relation to bone-related phenotypes in men and women. J Clin Densitom.

[28] Looker AC, Borrud LG, Hughes JP, Fan B, Shepherd JA, Melton LJ 3rd (2012) Lumbar spine and proximal femur bone mineral density, bone mineral content, and bone area:United States, 2005–2008. Vital Health Stat 11 251:14,44,46 24261130

[29] World Health Organization (2003) Prevention and management of osteoporosis. World Health Organ Tech Rep Ser 921:53–85. https://apps.who.int/iris/handle/10665/42841 15293701

[30] O’Leary TJ, Drake RB, Naylor JE (1972). The plaque control record. J Periodontol.

[31] Tonetti MS, Greenwell H, Kornman KS (2018). Staging and grading of periodontitis: framework and proposal of a new classification and case definition. J Periodontol.

[32] Charupinijkul A, Arunyanak S, Rattanasiri S, Vathesatogkit P, Thienpramuk L, Lertpimonchai A (2021) The effect of obesity on periodontitis progression: the 10-year retrospective cohort study. Clin Oral Investig:1–8. 10.1007/s00784-021-04031-2 10.1007/s00784-021-04031-2 34180000

[33] Sarafrazi N, Wambogo EA, Shepherd JA (2021) Osteoporosis or low bone mass in older adults: United States, 2017–2018. NCHS Data Brief:1–8. 10.15620/cdc:103477 34029181

[34] Tonetti M, Claffey N (2005). Advances in the progression of periodontitis and proposal of definitions of a periodontitis case and disease progression for use in risk factor research. Group C consensus report of the 5th European Workshop in Periodontology. J Clin Periodontol.

[35] Pei X, Ouyang X, He L, Cao C, Luan Q, Suda R (2015). A 4-year prospective study of the progression of periodontal disease in a rural Chinese population. J Dent.

[36] Needleman I, Garcia R, Gkranias N (2018). Mean annual attachment, bone level, and tooth loss: a systematic review. J Periodontol.

[37] Ogawa H, Yoshihara A, Hirotomi T, Ando Y, Miyazaki H (2002). Risk factors for periodontal disease progression among elderly people. J Clin Periodontol.

[38] Eickholz P, Kaltschmitt J, Berbig J, Reitmeir P, Pretzl B (2008). Tooth loss after active periodontal therapy. 1: patient-related factors for risk, prognosis, and quality of outcome. J Clin Periodontol.

[39] Penoni DC, Torres SR, Farias MLF, Fernandes TM, Luiz RR, Leão ATT (2016). Association of osteoporosis and bone medication with the periodontal condition in elderly women. Osteoporos Int.

[40] Passos-Soares JS, Vianna MIP, Gomes-Filho IS (2017). Association between osteoporosis treatment and severe periodontitis in postmenopausal women. Menopause.

[41] Penoni DC, Vettore MV, Torres SR, Farias MLF, Leão ATT (2019). An investigation of the bidirectional link between osteoporosis and periodontitis. Arch Osteoporos.

[42] Grossi SG, Zambon JJ, Ho AW (1994). Assessment of risk for periodontal disease. I. Risk indicators for attachment loss. J Periodontol.

[43] Löe H, Anerud A, Boysen H, Smith M (1978). The natural history of periodontal disease in man: the rate of periodontal destruction before 40 years of age. J Periodontol.

[44] Buchwald S, Kocher T, Biffar R, Harb A, Holtfreter B, Meisel P (2013). Tooth loss and periodontitis by socio-economic status and inflammation in a longitudinal population-based study. J Clin Periodontol.

[45] Qaseem A, Wilt TJ, Kansagara D (2018). Hemoglobin A1c targets for glycemic control with pharmacologic therapy for nonpregnant adults with type 2 diabetes mellitus: a guidance statement update from the American College of Physicians. Ann Intern Med.

[46] Demmer RT, Holtfreter B, Desvarieux M (2012). The influence of type 1 and type 2 diabetes on periodontal disease progression: prospective results from the Study of Health in Pomerania (SHIP). Diabetes Care.

[47] Xu S, Zhang G, Guo JF, Tan YH (2021). Associations between osteoporosis and risk of periodontitis: a pooled analysis of observational studies. Oral Dis.

[48] Lee JE, Kim KM, Kim LK (2017). Comparisons of TBS and lumbar spine BMD in the associations with vertebral fractures according to the T-scores: a cross-sectional observation. Bone.

[49] Padlina I, Gonzalez-Rodriguez E, Hans D (2017). The lumbar spine age-related degenerative disease influences the BMD not the TBS: the Osteolaus cohort. Osteoporos Int.

