# Supplementary material for: Decreased bone mineral density is associated with an increased number of teeth with periodontitis progression: a 5-year retrospective cohort study
Source: Clin Oral Investig. 2023 Dec 28;28(1):51. doi: 10.1007/s00784-023-05463-8 (PMC10754725; doi:10.1007/s00784-023-05463-8)
Supplement: Supplementary file 1 — Supplementary file1 (DOCX 31 KB) [file 784_2023_5463_MOESM1_ESM.docx]

**Supplementary Table 1.** Periodontal examination calibration (weight kappa ±1 mm)

|  | PPD | | RE/CAL | |
| --- | --- | --- | --- | --- |
|  | Inter-examiner | Intra-examiner | Inter-examiner | Intra-examiner |
| EGAT 1/5, 3/2 | 0.74–1.00 | 0.87–1.00 | 0.78–1.00 | 0.87–1.00 |
| EGAT 1/6, 3/3 | 0.74–1.00 | 0.86–1.00 | 0.72–1.00 | 0.91–1.00 |

Abbreviation: PPD, periodontal probing depth; RE, gingival recession; CAL, clinical attachment level.
