# Supplementary material for: Decreased bone mineral density is associated with an increased number of teeth with periodontitis progression: a 5-year retrospective cohort study
Source: Clin Oral Investig. 2023 Dec 28;28(1):51. doi: 10.1007/s00784-023-05463-8 (PMC10754725; doi:10.1007/s00784-023-05463-8)
Supplement: Supplementary file 2 — Supplementary file2 (DOCX 35 KB) [file 784_2023_5463_MOESM2_ESM.docx]

**Supplementary Table 2.** Risk ratios and 95% confidence intervals for an increased number of teeth with periodontitis progression

|  | Whole study population  (n = 2,400) | Population subgroups | | | | | |
| --- | --- | --- | --- | --- | --- | --- | --- |
|  |  | Postmenopausal women  (n = 304) | Non-  smokers  (n = 1,561) | Current/Former  Smokers  (n = 839) | Periodontitis stage | | |
|  |  |  |  |  | No/stage I  (n = 803) | Stage II  (n = 415) | Stage III/IV  (n = 1,182) |
| Adjusted RR (95% CI) for an increased number of teeth with periodontitis progression^a,b,c^ |  |  |  |  |  |  |  |
| Osteoporosis | 1.08 (0.97–1.19) | 1.31 (1.09–1.58)^*^ | 1.19 (1.04–1.36)^*^ | 0.92 (0.77–1.10) | 1.05 (0.84–1.31) | 0.70 (0.47–1.06) | 1.13 (1.00–1.28)^*^ |
| Adjusted RR (95% CI) for an increased number of teeth with an additional proximal CAL loss of ≥3 mm over 5 years^a,d^ |  | | | | | | |
| Osteoporosis | 1.03 (0.91–1.17) | 1.11 (0.88–1.39) | 1.09 (0.94–1.28) | 0.91 (0.73–1.14) | 0.90 (0.70–1.16) | 0.69 (0.45–1.04) | 1.14 (0.97–1.33) |
| Adjusted RR (95% CI) for an increased number of tooth loss with baseline proximal CAL ≥5 mm^a,e^ |  | | | | | | |
| Osteoporosis | 1.35 (1.10–1.67)^*^ | 1.99 (1.30–3.04)^*^ | 1.90 (1.42–2.52)^*^ | 0.97 (0.69–1.35) | N/A | N/A | 1.25 (0.99–1.56) |

Abbreviation: BMD, bone mineral density.

^a^Risk ratios and 95% confidence intervals were obtained using Poisson regression analysis.

^b^Periodontitis progression was defined as a tooth presenting an additional proximal CAL loss of ≥3 mm or an additional lost tooth with a baseline proximal CAL ≥5 mm.

^c^Adjusted by sex, age, baseline periodontal status, plaque score, diabetes, smoking, self-report periodontal treatment, education.

^d^Adjusted by sex, age, baseline periodontal status, plaque score, diabetes, BMI, smoking, self-report periodontal treatment, education.

^e^Adjusted by sex, age, plaque score, diabetes, BMI, smoking, education. (baseline periodontal status was not included as a covariable, and the analysis in no/stage I and stage II periodontitis subgroups were not performed due to the limited number of tooth loss with baseline proximal CAL ≥5 mm).

^*^Significant difference (*P* <0.05)
